# Economic Analysis of Delivering Primary Health Care Services through Community Health Workers in 3 North Indian States

**DOI:** 10.1371/journal.pone.0091781

**Published:** 2014-03-13

**Authors:** Shankar Prinja, Gursimer Jeet, Ramesh Verma, Dinesh Kumar, Pankaj Bahuguna, Manmeet Kaur, Rajesh Kumar

**Affiliations:** 1 School of Public Health, Post Graduate Institute of Medical Education and Research, Chandigarh, India; 2 Department of Community Medicine, Pt. BD Sharma Post Graduate Institute of Medical Sciences, Rohtak, Haryana, India; 3 Department of Community Medicine, Dr. Rajendra Prasad Government Medical College, Kangra, Himachal Pradesh, India; University of Alabama at Birmingham, United States of America

## Abstract

**Background:**

We assessed overall annual and unit cost of delivering package of services and specific services at sub-centre level by CHWs and cost effectiveness of Government of India’s policy of introducing a second auxiliary nurse midwife (ANM) at the sub-centre compared to scenario of single ANM sub-centre.

**Methods:**

We undertook an economic costing of health services delivered by CHWs, from a health system perspective. Bottom-up costing method was used to collect data on resources spent in 50 randomly selected sub-centres selected from 4 districts. Mean unit cost along with its 95% confidence intervals were estimated using bootstrap method. Multiple linear regression model was used to standardize cost and assess its determinants.

**Results:**

Annually it costs INR 1.03 million (USD 19,381), or INR 187 (USD 3.5) per capita per year, to provide a package of preventive, curative and promotive services through community health workers. Unit costs for antenatal care, postnatal care, DOTS treatment and immunization were INR 525 (USD 10) per full ANC care, INR 767 (USD 14) per PNC case registered, INR 974 (USD 18) per DOTS treatment completed and INR 97 (USD 1.8) per child immunized in routine immunization respectively. A 10% increase in human resource costs results in 6% rise in per capita cost. Similarly, 10% increment in the ANC case registered per provider through-put results in a decline in unit cost ranging from 2% in the event of current capacity utilization to 3% reduction in case of full capacity utilization. Incremental cost of introducing 2^nd^ ANM at sub-centre level per unit percent increase ANC coverage was INR 23,058 (USD 432).

**Conclusion:**

Our estimates would be useful in undertaking full economic evaluations or equity analysis of CHW programs. Government of India’s policy of hiring 2^nd^ ANM at sub-centre level is very cost effective from Indian health system perspective.

## Introduction

Evidence on cost of health care delivered through community health workers (CHWs) is useful for public health planning, monitoring and evaluation. With the onset of decentralized planning process in a number of low and middle income countries, there is a need for generating reliable estimates of unit cost for various services to aid in planning [1], [2]. Moreover, with increased advocacy on scale up of CHWs, these cost estimations become all the more important [3]–[5]. Secondly, unit cost of service delivered by health workers is needed by researchers assessing the technical and allocative efficiency [6]. Hence, cost information is required not only for fiscal planning, but also for making the right choices which yield the best value for money spent on health care.

In terms of research and evaluation studies which draw upon the cost data are a range of economic evaluations. Unit cost data is also needed by those engaged in equity research, to estimate the extent of distributional benefits of public spending [7]. Lack of unit cost data on health service provision at different levels has been cited as a limitation by researchers in undertaking a complete benefit incidence analysis [8], [9].

Economic evaluation of CHWs is even more important from Indian health system viewpoint. In India, there are 1,48,366 sub-centres which is the grassroots level health care institution for provision of primary care services delivered by community health workers (CHW) [10]. Under the National Rural Health Mission, there is significant emphasis on augmenting the availability of CHWs at sub-centres. The number of ANMs at Sub Centres and PHCs have increased from 133194 in 2005 to 207578 in 2012 which amounts to an increase of about 56% [10]. Hence it is very important to assess the economic implications of such extensive scale-up by investing in CHW programs.

Despite apparent need for evidence on cost of service provision from a health system perspective, available evidence is inadequate. The existing evidence is mainly drawn from a few sources. Firstly, the National Health Accounts (NHA), which have been institutionalized in a number of countries, provides health system expenditure on different levels of care – primary, secondary and tertiary; by financing sources, agents and providers. [11]. However, the entire exercise of NHA is grounded in estimation of financial costs, rather than a more complete economic costing. Thus NHA measures *volume of expenditure* rather than the *value* of economic activities. This has implications for measuring capital resources as NHA measures only capital expenditure during the year of measurement irrespective of the capital resources being used to delivery services [12]. Second practical drawback of NHA in low and middle income countries is non-availability of good data sources to value donated goods and services [12], [13]. The other source for health system costs at CHW level are findings of the WHO-CHOICE study [14]. While this study has sound methodological basis, questions of generalizing South East Asian Region (SEAR) estimates to India is usually questioned. Moreover, the WHO-CHOICE estimates are almost seven year old for application today. Similarly, the estimates of the National Commission on Macroeconomics and Health (NCMH) are also dated [15]. Finally, there is limited published literature on health system cost of service provision through community health workers [16]. Most of these studies are focal in nature; focal in terms of the geographical representation of sample coverage or the range of services which are covered for costing.

In a review of effectiveness of CHWs, it was found that these workers are beneficial in terms of improving immunization coverage and outcomes for malaria and acute respiratory infections as compared to usual care [17]. These programmes are expected to improve the cost-effectiveness of health care systems by reaching large numbers of previously underserved people with high-impact basic services at low cost [18]. However, a review of evidence on cost effectiveness of CHW programs found that the while most studies find a positive impact of CHW programs on coverage and equitable utilization than alternative modes of program delivery, majority of the studies do not evaluate the economic impact of CHW programs comprehensively [19]. Moreover, whatever evidence exists, is focused on specific programs or services [16], with little evidence on the cost effectiveness of CHWs in provision of the gamut of services or platform of care which they provide. A more recent systematic review which included studies on CHWs from 1980 to 2006 found only 6 studies which estimated the cost or cost effectiveness of CHW programs [20]. Even this review concluded that whatever studies exist are not full economic evaluations and are inadequate to make meaningful conclusions. In view of this lack of a robust evidence, WHO invited for proposals on assessment of cost effectiveness of CHW programs [21].

We aim to bridge this gap in literature in this paper where we report the cost of delivery of a platform of health care services delivered through the CHWs at sub-centre (SC) level. We also estimate the unit cost of specific services, and the contribution of different programs to cost of providing health care services at sub-centre level. Finally, we analysed the cost effectiveness of Government of India’s policy of hiring a 2^nd^ ANM at sub-centre level as compared to traditional single ANM sub-centres. This would help in planning the cost of scale-up of current community health worker programs.

## Methods

### Study Setting and Service Platform

We undertook this study in 3 North Indian states of Haryana, Punjab and Himachal Pradesh. Together, these states comprise of nearly 60 million Indian population. We randomly selected a total of 4 districts from Haryana (2), Punjab (1) and Himachal Pradesh (1). In these 4 districts, we randomly selected 50 sub-centres –24 in Haryana, 11 and 15 each in Punjab and Himachal Pradesh respectively.

A sub-centre is the lowest level of health institution in India, which caters to the health needs of about 5000 population. It is manned by an Auxiliary Nurse Midwife (ANM) and a male Multi-Purpose Health Worker (MPHW (M)). Since the launch of National Rural Health Mission, a new cadre of community health workers – accredited social health activist (ASHA) was added in each village [22]. ASHAs were created at village level to act as a social change agent to generate demand for health care services, through awareness generation and behaviour change communication among the community. Secondly, additional ANMs were recruited at sub-centre level to share the workload of the existing ANMs. An ANM at sub-centre level provides antenatal care, natal care, postnatal care, immunization and family planning services. Moreover, ANM provides curative care for minor illnesses such as fever, diarrhoea and acute respiratory illness; and referral for serious illness. Other services provided at sub-centre, primarily by MPHW (M), includes disease surveillance, monitoring water quality and health education. Besides these services, sub-centre staff is involved in record keeping and attending review meetings. We estimated the cost of this platform of services provided at sub-centre level comprised by the team of ANMs, MPHW (M) and ASHA workers.

As per recent Indian Public Health Standards, there are 2 types of sub-centres – type A and B (MCH-sub-centres) [23]. Both types of sub-centres have 1 post of MPHW (M). While type A sub-centre requires 1 mandatory (and 2 desirable) ANM, there are 2 mandatory ANMs which are recommended for type B sub-centres. Further, in type B sub-centres, there is provision for a staff nurse or a 3^rd^ ANM. Nearly 4–6 ASHA workers exist per sub-centre.

### Cost Data Collection

Economic cost of services was assessed from a health system perspective, using a bottom-up costing methodology [24], [25]. Cost centres at sub-centre were identified in terms of service centres and support centres. Data on all resources, capital and recurrent, consumed for provision of all services during the financial year 2012 (April 2012 to March 2013) was collected. Routine records at the sub-centre level (such as stock register, monthly reports etc.) were used to collect the data. This was supplemented with data on incentives paid under various health schemes (to service providers or beneficiaries in case of conditional cash transfers), untied funds and annual maintenance grants was collected from the office of Civil Surgeon at district level. Facility survey of sub-centre was undertaken to assess the capital resources, i.e. building. Non-consumable stock register was reviewed for number of equipment and other capital goods. This was supplemented by physical observation of facility.

All the staff members at the sub-centre, were interviewed using a semi-structured interview schedule on time allocation for different services. While we do acknowledge presence of more robust time-motion studies to understand the time allocation patterns [26], however, activity patterns at sub-centre preclude the application of such methods. Although time motion studies provide valid estimates of time spent on different activities, such observations are usually conducted over a finite period of time, which usually ranges over a period of few weeks. This observation may be repeated over different months to account for seasonal variations. However, many activities at sub-centre level such as mass polio immunization campaigns, school health check-up, household survey etc. occur for a few days per year and hence are likely to be missed in such observation based methods. We developed and used a tool which captured details of all such activities which are undertaken at different frequencies such as once or twice a year, once a quarter, monthly or weekly, besides daily activities; and time spent when the activity was carried out. Fixed-time equivalents of each staff were estimated.

Data on services provided and other demographic details of the population covered was collected from routine monthly reports and respective service registers. The study was approved by the Institute Ethics Committee of the Post Graduate Institute of Medical Education and Research, Chandigarh, India. We took administrative approval of Department of Health, of respective State Governments and the Civil Surgeons of respective districts. Written informed consent was taken to interview staff for time allocation.

### Cost Data Analysis

Cost of building space was estimated by multiplying the floor area with the prevailing market rental price of land. The annualized cost of other capital equipment and goods was estimated after adjustment for the useful life of the equipment and a discount rate of 3% [26], [27]. All prices of the equipment were converted to current price in 2012 using the prevailing GDP deflators. All costs were converted to US dollars at an exchange price of INR 53.3 per single US dollar [28]. Average market prices for drugs, consumables and equipment were used instead of the price at which the Government procured the same as Government could be acting as a monopsonistic purchaser and distorting the market. For time cost of human resources, we used the salaries paid, as there is no private market for the ANMs, MPHW (M) or ASHA workers which could have provided ‘shadow prices’. Unit cost of service provision at sub-centre level was defined as the cost of providing the platform of all services per year per person covered under the sub-centre. It was computed as a ratio of the value of all resources spent on provision of care during a year and the total population served by sub-centre.

Overall cost of delivery of platform of health services at sub-centre level was assessed by the type of services i.e. preventive, curative, promotive etc.; or outpatient, inpatient (delivery), outreach etc.; and by type of program i.e. maternal health, child health, family planning etc. Certain resources, i.e. capital and recurrent were used solely for specific types of services or programs, and were hence allocated totally for specific service or program. However, most of the resources, especially those which were capital in nature, were shared between different programs and services. Such joint costs were apportioned into cost for different services or programs using appropriate statistics. For example, the capital cost for room (or equipment or category of medicine/consumable etc.) shared for provision of different services or programs was apportioned between different programs by the proportion of time used by clients for a particular service or program. This indicator for apportionment combined the effect of the number of clients for a particular service and the time spent on each client for that service, and is superior to an indicator which merely considers the proportion of clients for a given service to apportion cost.

We also estimated the unit cost of provision of specific services such as antenatal care per pregnant woman registered, postnatal case per mother registered, per immunization injection in routine session or as part of intensive pulse polio immunization (IPPI) campaign, per tubectomy case motivated, per patient treatment completed with directly observed short-course (DOTS) chemotherapy. Unit costs were also estimated per outpatient client consultation and cost of providing platform of child health services per child per year.

To increase the robustness of estimates, we estimated median estimate of unit cost and its 95% confidence limits using bootstrap method in which individual cost heads for a given sub-centre were randomly simulated over 1000 times. In order to adjust for varying capacity utilization, we adjusted the unit cost estimates for various services at 80% and 100% capacity utilization. Since the services delivered by CHWs at sub-centre level are very heterogeneous, we used the ANC coverage as the proxy indicator to adjust for capacity. In order to examine the determinants of cost of community health worker health services, we undertook multiple linear regression to identify the predictors and their predictive power taking cost per person per year as dependent variable. The different models developed for standardization of parameter estimates were based on the equation mentioned below:


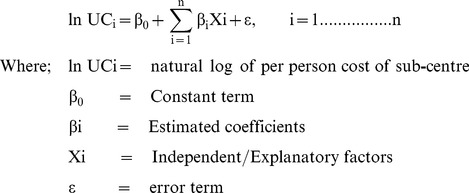
(1)

Human resource costs, population catered and ANC cases registered per CHW at sub-centre were taken as independent predictors. Log transformation was undertaken for dependent and independent variables to reduce the skewness and number of outliers; and to improve normality, linearity and homoscedasticity of residuals. Number of deliveries (more than or less than 30 per month) in sub-centre was introduced as dummy variable in the regression. Beta coefficients for each factor along with its standard error are reported in the results. Cost per person per year, adjusted at 80% and 100% capacity utilization, were included as dependent variable in two separate models respectively. Goodness-of-fit of the model was tested using a variety of measures such as heteroskedasticity, multicollinearity, R^2^ and F-test. Multicollinearity was tested using tolerance test and variance inflation factors (VIF).

### Cost Effectiveness Analysis

From a health system perspective, we analysed the cost effectiveness of Government of India’s policy of introducing a 2^nd^ ANM at the sub-centre level. The benefits were measured in terms of increase in coverage of antenatal care (ANC) coverage. Incremental cost effectiveness ratio (ICER) was computed to represent incremental cost of introducing 2^nd^ ANM per unit increase in ANC coverage, as compared to sub-centres served by 1 ANM.

## Results

A total of 50 sub-centres were covered in the three districts i.e. 24 in Rohtak and Panchkula districts of Haryana state, 15 in Kangra district of Himachal Pradesh and 11 in Fatehgarh district of Punjab. Nearly 44% sub-centres had one ANM in position, while 52% sub-centres had two ANMs. One sub-centre in Punjab had 3 ANMs to provide the services. The average population catered by each sub-centre was approximately 6000. A total of 17 (34%) sub-centres catered to a population of less than 5000, 50% sub-centres provided services to population ranging from 5000 to 7500, while 8 (16%) centres served more than 7500 population. Reported average ANC coverage for sub-centres was 73% with a range of 46% to 100%. There were 16 (32%) sub-centres with a delivery load of more than 30 per month.

### Overall Cost of Service Delivery

**Figure 1:**
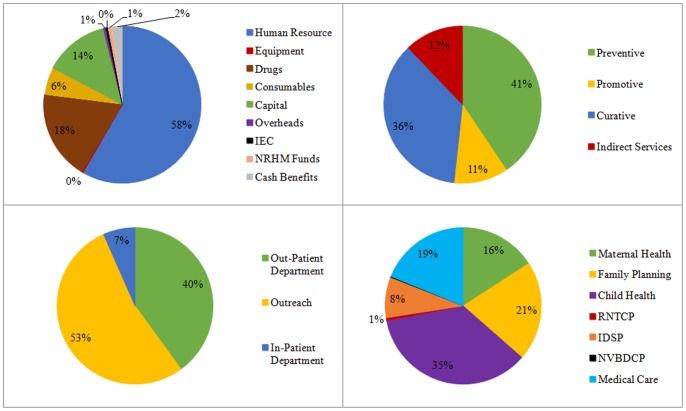
Proportional distribution of cost of health service delivery through community health worker.

Median costs of providing a package of services through community health workers at sub-centre along with the 95% confidence interval presented in Table 1. Overall it costs nearly INR 1.03 million (USD 19,381) per year to provide health services through CHWs at sub-centre. Cost of human resource alone accounts for the 58%, followed by drugs (18%) and capital (13%) (Table 1, Figure 1). Forty one percent of the cost incurred was for delivering preventive services, 36% for curative care, while remaining 23% was used for providing promotive or other indirect services (Table 2). Almost half of the cost was incurred in provision of services as part of an outreach program while 40% resources were spent in delivering services in out-patient setting. Institutional deliveries, which was the only inpatient service offered at sub-centre, accounted for 7% of the total cost. Majority cost incurred at the level of community health workers was on the programs targeting under-5 children (36%), family planning (21%) and maternal health (16%). (Table 2).

**Table 1:** Cost of delivering health care services at sub-centre level, India.

Cost Head	Median INR (USD)	2.5th Percentile INR (USD)	97.5th Percentile INR (USD)
Human Resource	602737 (11308)	544835 (10222)	657212 (12330)
Equipment	3157 (59)	2711 (51)	3645 (68)
Drugs	189417 (3554)	159226 (2987)	225344 (4228)
Consumables	59199 (1111)	46457 (872)	74393 (1396)
Capital	138329 (2595)	97878 (1836)	48681 (913)
Overheads	5575 (105)	4128 (77)	183913 (3451)
IEC	3443 (65)	2394 (45)	4845 (91)
NRHM Funds	8204 (154)	6924 (130)	9451 (177)
Cash Benefits	22970 (431)	16319 (306)	33431 (627)
**Total**	**1033031 (19381)**	**880872 (16527)**	**1240914 (23282)**

**Table 2:** Cost of delivering health care services at primary and secondary level by type and level of service and type of program, India.

Costs	Median INR (USD)	2.5th Percentile INR (USD)	97.5th Percentile INR (USD)
***Level of Service***			
Preventive	416869 (7821)	373367 (7005)	457249 (8579)
Promotive	114496 (2148)	86580 (1624)	145547 (2731)
Curative	370991 (6960)	324757 (6093)	417494 (7833)
Indirect Services	124452 (2335)	99616 (1869)	156450 (2935)
***Nature of Service Provided***			
Out-Patient Department	326760 (6131)	283195 (5313)	375329 (7042)
Outreach	436253 (8185)	384303 (7210)	486480 (9127)
In-Patient Department	53872 (1011)	29337 (550)	79190 (1486)
***Type of Program***			
Maternal Health	163058 (3059)	145226 (2725)	180838 (3393)
Family Planning	212303 (3983)	184709 (3465)	238997 (4484)
Child Health	365559 (6859)	331428 (6218)	396927 (7447)
RNTCP	5563 (104)	4090 (77)	7421 (139)
IDSP	86874 (1630)	62136 (1166)	113920 (2137)
NVBDCP	1840 (35)	1175 (22)	2563 (48)
Medical Care	193525 (3631)	158501 (2974)	229503 (4306)

Note: RNTCP (Revised National TB Control Program), IDSP (Integrated Disease Surveillance Program), NVBDCP (National Vector Borne Diseases Control Program).

### Unit Costs

We found that in order the health system incurs a cost of INR 187 (95% CI: 166–209, USD 3) per capita per year to provide a package of preventive, curative and promotive services through community health workers at sub-centre level (Table 3). At 80% and 100% capacity utilization, cost per person per year increased to INR 198 (USD 3.7) and INR 215 (USD 4) respectively. Unit costs are higher for providing services like polio immunization (INR 1474 or USD 28 per child), school health program (INR 1078 or USD 20 per child examined) and DOTS treatment (INR 974 or USD 18 per patient treatment completed). Average cost of providing 1 full antenatal care to a pregnant woman and post natal care per mother is INR 525 (USD 10) and INR 767 (USD 14) respectively. (Table 3).

**Table 3:** Unit Cost of providing health care services through Community Health workers (CHW) in India.

Unit Cost	Unadjusted INR (USD)			Adjusted at 80% capacity utilization INR (USD)			Adjusted at 100% capacity utilization INR (USD)		
	Median	Percentile		Median	Percentile		Median	Percentile	
		2.5^th^	97.5^th^		2.5^th^	97.5^th^		2.5^th^	97.5^th^
Per person per year	187 (3.5)	166 (3.1)	209 (3.9)	199 (4)	176 (3)	220 (4)	216 (4)	192 (4)	239 (4)
Per child per year	576 (11)	519 (10)	640 (12)	613 (12)	550 (10)	674 (13)	666 (12)	600 (11)	733 (14)
Per out-patient consultation	370 (7)	299 (6)	465 (9)	394 (7)	317 (6)	490 (9)	428 (8)	345 (6)	532 (10)
Per ANC case registered	525 (10)	456 (9)	619 (12)	559 (10)	483 (9)	652 (12)	607 (11)	526 (10)	708 (13)
Per PNC case registered	767 (14)	538 (10)	1092 (20)	817 (15)	570 (11)	1150 (22)	887 (17)	621 (12)	1250 (23)
Per child immunized (Routine)	97 (1.8)	77 (1.5)	120 (2.3)	103 (2)	82 (2)	127 (2)	112 (2)	89 (2)	138 (3)
Per child immunized (IPPI)	1474 (28)	67 (1.3)	3273 (61)	1570 (29)	71 (1)	3447 (65)	1704 (32)	77 (1)	3748 (70)
Per child examined (School Health)	1078 (20)	375 (7)	1951 (37)	1148 (22)	398 (7)	2055 (39)	1247 (23)	433 (8)	2234 (42)
Per Tubectomy motivation (Family Planning)	561 (11)	347 (7)	887 (17)	598 (11)	368 (7)	934 (18)	649 (12)	401 (8)	1016 (19)
Per patient complete DOTS treatment	974 (18)	753 (14)	1249 (23)	1037 (19)	798 (15)	1316 (25)	1126 (21)	870 (16)	1430 (27)

Note: ANC (antenatal care), PNC (postnatal care), IPPI (Intensive Pulse Polio Immunization), DOTS (Directly Observed Treatment Short-term for TB patients).

### Determinants of Cost of Health Services

Our regression model explained nearly 78% to 82% variability in the estimate for cost per person per year, depending on the capacity utilization. The tolerance value and VIF ranged between 0.535–0.845 and 1.18–1.86 respectively indicating absence of multicollinearity. Controlling for other determinants, we found that a 10% increase in human resource cost leads to a 6% (p<0.001) increase in the cost per person per year (Table 4). Similarly, 10% increment in the ANC case registered per provider through-put results in a decline in unit cost ranging from 2% (p<0.05) in the event of current capacity utilization to 3% (p<0.01) reduction in case of full capacity utilization. Finally, the unit cost estimates declined by about 5% with a 10% increase in the population (p<0.001).

**Table 4:** Association of different parameters with cost per person at sub-centre level.

Variables	β coefficients (Standard Error)#		
	Model A	Model B	Model C
**Constant**	2.7 (1.3)	3.4 (1.2)**	3.4 (1.2)**
**Ln Human Resource Costs**	0.6 (0.08)***	0.6 (0.07)***	0.6 (0.07)***
**Ln ANC registered per provider**	−0.2 (0.09)*	−0.2 (0.09)*	−0.3 (0.09)**
**Ln Population catered**	−0.5 (0.1)***	−0.6 (0.09)***	−0.5 (0.09)***
**Dummy (More than 30 deliveries in a month)**	−0.03 (0.07)	0.02 (0.06)	0.05 (0.06)
**R^2^**	.78	.82	.82
**Adjusted R^2^**	.76	.80	.80

*p<0.05, **p<0.01, ***p<0.001.

# **Note**: Model A-Dependent variable is natural log of unadjusted cost per person per year.

Model B- Dependent variable is natural log of cost per person per year adjusted at 80% capacity utilization.

Model C- Dependent variable is natural log of cost per person per year adjusted at 80% capacity utilization.

### Cost Effectiveness of 2^nd^ ANM Policy

Sub-centres with 1 ANM served an average population of 4697 persons and registered mean number of 79 pregnant women for antenatal care (Table 5). Similarly sub-centres with 2 ANM catered to an average population of 7067 and registered about 141 pregnant women. The coverage of ANC care in sub-centre with 1 and 2 ANMs ranged from 68% to 80% respectively.

**Table 5:** Characteristics and cost estimates for CHW services among sub-centres with one and two Auxiliary Nurse Midwife.

Characteristics	One ANM sub-centres			Two ANM sub-centres		
	Mean	95% Confidence Interval		Mean	95% Confidence Interval	
		LL	UL		LL	UL
Population	4697	4163	5247	7062	5976	8457
ANC registered	79	70	89	141	117	172
ANC coverage (%)	68	63	72	80	71	90
Total Annual cost	864312	773399	959393	1151604	1023532	1271182
INR per person (per year)	196	168	226	178	150	210
INR per ANC registered	582	470	719	485	377	609
INR per PNC registered	1212	735	1815	345	240	473
INR per child immunized	89	66	116	103	73	139

*ANM: Auxiliary Nurse Midwife, ANC: Antenatal case, PNC: Postnatal case, LL: Lower limit, UL: Upper limit.

The annual cost varied from INR 0.86 (95% CI: 0.77–0.95) million for sub-centres with 1 ANM; to INR 1.15 (95% CI: 1.08–1.27) million per year for sub-centres with 2 ANMs. There was statistically insignificant difference in per person per year costs between sub-centres with 1 ANM (INR 178; 95% CI: 150–210) and sub-centre with 2 ANMs (INR 196; 95% CI: 168–226) (Table 5). Incremental cost of introducing 2^nd^ ANM at sub-centre level is INR 23058 (USD 432.6) per unit increase in ANC coverage.

## Discussion

We undertook this study in North India to bridge a gap in existing literature on cost and cost effectiveness of community health workers. In our analysis of 50 sub-centres, we found that annually it costs nearly INR 1.03 million (USD 19,381), with a unit cost of INR 187 (USD 3.5) per capita per year, to provide a package of preventive, curative and promotive services through community health workers. Salary of human resources constitute the major proportion (60%) of cost of service delivery at this level. Services are primary delivered in an outreach mode –53% of total cost. In terms of health programs, child health (36%), maternal health (16%) and family planning (21%) constituted major costs. Unit costs were also reported for provision of specific services such as ANC care (INR 525 per full ANC care provided), postnatal care (INR 767per 1 women delivered care), DOTS case treatment completed (INR 974) etc. The value of incremental cost effectiveness ratio value of USD 432 is much below the GDP per capita of India (INR 68, 747 per capita, USD 1290 [29]) reflects that the introduction of 2^nd^ ANM at sub-centres is very cost effective from Indian health system viewpoint [27].

As per our knowledge, this is the first study from India which comprehensively estimates the cost of delivery of entire package of services by the CHWs at sub-centre level. Our estimates represent the economic costs or opportunity cost of resources spent for provision of services delivered by CHWs. There are a few studies which specifically estimate the cost of focused services – such as child health services, or from a focused geographic region [16]. Secondly, we present the results from the perspective of planning needs for scale-up of different services, such as maternal health, child health etc. We also estimated the unit costs of most important services delivered by CHWs which can be used in future economic evaluations for estimating cost effectiveness or cost utility of health workers; and in assessing the distributional benefits of CHW programs in a benefit incidence analysis.

National Commission on Macroeconomics and Health (NCMH), in 2005, undertook a detailed estimation of cost of health services in India. They reported the annual costs of delivering health services at sub-centre level as INR 799,010 (USD 14,991). Adjusting for the effect of inflation at the rate of 8.23% during the intervening year, the NCMH estimated cost in real terms would be INR 1.43 million in 2012–13. Similarly, the inflation adjusted values for unit cost per ANC and PNC were INR 484 and INR 412 respectively. Difference in annual cost and unit costs between those reported in our study and NCMH estimates could be on account of a number of factors. Firstly, the NCMH estimates are about 8 years old at a time when the National Rural Health Mission (NRHM) was not initiated [22]. Since the onset of NRHM, decentralized planning has led to devolution of higher funding at lower levels [2]. Secondly, new cadre of human resources at sub-centre level have been added – accredited social health activist (ASHA) and additional ANM. Thirdly, the coverage of various RCH services have also increased since the NRHM [30], [31]. There are some interesting differences between the inflation adjusted NCMH estimates and our findings. For example, while the overall costs reported in NCMH appears to be slightly higher than our finding, unit cost of provision of antenatal care as per our study is almost similar (8% higher in our study), and postnatal care is 86% higher. Difference in overall cost could be as a result of the fact that majority of the sub-centres in our sample did not have the MPHW (M). As a result the overall cost is lesser than NCMH estimate. However, unit costs for maternal and child health services in our study are either higher or similar. This could be explained on the basis of an increase in the coverage and scope of ANC and PNC services. A new cadre of ASHA workers were incorporated in the system whose payments are linked with coverage of home-based postnatal care. There is better availability of services such as drugs (iron folic acid tablets), weighing scales and diagnostic equipment for testing blood and urine samples during pregnancy. As a result of these, proportionate cost for ANC and PNC services increased post-NRHM which is reflected in our study.

Although we found that the overall annual cost was slightly higher for sub-centres with 2 ANMs, there were statistically insignificant difference in total annual and per person annual costs between the sub-centres with 1 or 2 ANMs. This could be as a result of two features. Firstly, the second ANM is not paid as much as the regular 1^st^ ANM. Hence the marginal costs of hiring 2^nd^ ANM is not as high. Secondly, the presence of 2^nd^ ANM results in improvement in coverage, hence more persons are able to obtain service or more pregnant women reached out to provide full antenatal care. As a result the unit costs do not rise. This has significant implications for India’s policy of hiring a 2^nd^ ANM, i.e. although it appears that the second ANM puts a fiscal pressure on the system, however, the increase in service coverage and the low marginal cost implies that the same is value for money. Government of India’s policy of hiring 2^nd^ ANMs at sub-centre under the National Rural Health Mission is very cost effective (ICER = USD 423) and thus justified on economic grounds. Finally, it also implies that the Government of India should also consider introducing some reforms in the way human resources for health are paid such that it improves the efficiency of the health system. These payment reforms could include a system in which part salary is paid as fixed amount while the remaining is paid as a performance based incentive. This has its own set of caveats and hence will require careful designing such that it does not crowd-out services which are not measured as part of performance assessment.

Moreover, studies published by the Commission on Macroeconomics and Health, the United Nations Millennium Project and the International Task Force on Innovative Financing for Health, in a low-income country a primary-health-care system should cost from US$ 50 to US$ 55 per capita per year in 2011 prices [32], [33]. In view of this, our estimates suggest that the CHW platform in the 3 North Indian states is costing nearly 5.5% to 6% of the total costs expected of the overall primary health care services delivery system. So there needs to be more investment in the CHW platform through sub-centres as the outcomes of such services delivered in an outreach mode have been found to be very equitable.

In an earlier analysis published recently, we had reported the cost of delivery of child health services in two alternate scenarios – with and without IMNCI program [16]. This study reported annual per child cost of INR 472 (USD 10.5) for ANM and INR 170 (USD 7.8) at ASHA level. We estimated the cost of the platform comprising of both ANM and ASHA and found the annual per child cost of INR 576 (USD 10.8). Both the previous and current study found that salary of human resources is the single most important constituent of the overall cost of health care. This finding about human resource cost is validated by a number of other costing studies worldwide [34]. Finally, our observation that 1% increase in volume of work reduces the unit cost by 0.2%–0.3% (depending on capacity utilization), is similar to results of an earlier analysis which reported a 0.27% reduction in the cost per outpatient visit with a 1% increase in number of patients [35].

Our study has some methodological limitations. Firstly, we adjusted all unit cost estimates in our study for capacity utilization using a single indicator, i.e. ANC coverage. While it might be appropriate to use specific indicators for capacity utilization for each service, the nature of services delivered by CHWs at sub-centre level preclude such opportunity. The services delivered by CHWs are heterogeneous. Any idle capacity from one service can be easily utilized by engaging in another activity. In such a situation, ANC service coverage, which also happens to be one the mainstay activity, allows a reasonable opportunity to estimate the extent of capacity utilization. Secondly, we did not undertake a full economic evaluation. We report the benefits in terms of number of services delivered, i.e. ANC check-ups or DOTS treatment completed. In an ideal scenario, it will be appropriate to compare the costs and benefits of the delivery of health services through CHWs with alternative methods of delivery (or compare different cadre of CHWs), and report the benefits in terms of more accepted utility measures such as a disability adjusted life year (DALY) or a quality adjusted life year (QALY). We also recognize that the mathematical specification of the model we report here, the log-log form, does not allow the identification of diseconomies of scale if they exist. Thirdly, we acknowledge that there are wide variations in the districts in India, in terms of resources used at sub-centre level, type of services delivered and in the coverage of services delivered. In order to test the variability of our estimates, we undertook stratified analysis for total cost, per person per year cost, and unit costs for specific services by the type of state. Comparing the cost estimates separately for Haryana, Punjab and Himachal Pradesh, we found statistically insignificant difference across the states. However, our estimates of cost may not be generalizable to All-India level. The relationship of unit cost with other determinants as shown in our analysis could be used for such estimations as the same explains about 80% of variation in the cost. We concede not having measured the indirect costs of service utilization which were borne by the households in the form of any out-of-pocket costs or productivity losses. However, we believe that such costs even if they exist for preventive services, would be very minor. Finally, a measurement of long term outcomes such as a disability adjusted life year (DALY) would be more useful for making a robust conclusion on cost effectiveness which can also be used to make comparisons across programs.

We acknowledge that in the true sense, human resource costs goes into the calculation of the unit cost, and hence its inclusion as an independent predictor could be questioned. However, the case of costing at the sub-centre level is different. In case of sub-centres, number of CHWs is determined by norms. Hence for every sub-centre, an ANM is posted irrespective of the volume of output. Plus, an additional ANM on contract basis is hired under the National Rural Health Mission. Hence unlike any other health institution, human resource cost behaves as a fixed cost at the sub-centre level and does not vary with the volume of services which are delivered. In our sample of sub-centres, the contribution of the human resource cost as a proportion of total sub-centre cost varied from 25% to 82%. So we consider that its inclusion as one of the independent variables is justified. The same methodology for regression has also been used and recommended by the WHO-CHOICE study [35].

Overall, we conclude that community health workers offer a low-cost option for delivery of health care services. Our study provides important evidence on cost of delivery of CHW health services, which can be used for future full economic evaluations to assess the overall cost effectiveness of delivery of platform of services at CHW level; or estimating the cost effectiveness of different programs delivered by CHWs. Our results also validate from an economic viewpoint, Government of India’s move to introduce a 2^nd^ ANM and its payment mechanism. We also provide the necessary evidence to plan for scale-up of CHW health worker programs which is the central strategy for meeting the MDGs in most low and middle income countries [5]. Evaluating the cost effectiveness of placing 2^nd^ ANM in sub-centres, using more long-term health outcomes such as DALYs averted is recommended as a future area of research.
